# Entropy Analysis of Magnetized Carbon Nanofluid over Axially Rotating Stretching Disk

**DOI:** 10.3390/ma15238550

**Published:** 2022-11-30

**Authors:** Hossam A. Nabwey, Uzma Sultana, Muhammad Mushtaq, Muhammad Ashraf, Ahmed M. Rashad, Sumayyah I. Alshber, Miad Abu Hawsah

**Affiliations:** 1 Department of Mathematics, College of Science and Humanities in Al-Kharj, Prince Sattam bin Abdulaziz University, Al-Kharj 11942, Saudi Arabia; 2 Department of Basic Engineering Science, Faculty of Engineering, Menoufia University, Shebin El-Kom 32511, Egypt; 3 Department of Mathematics, COMSATS University Islamabad, Islamabad 44000, Pakistan; 4 Department of Mathematics, University of Sargodha, Sargodha 40100, Pakistan; 5 Department of Mathematics, Faculty of Science, Aswan University, Aswan 81528, Egypt

**Keywords:** carbon nanofluid, magnetohydrodynamics, Joule heating, entropy, rotating stretching disk, carbon nanotubes

## Abstract

Nanofluids receive recognition from researchers and scientists because of their high thermal transfer rates. They have impactful industrial and technological modules in daily activities. In recent times, the heat transfer rate has been strengthened even more by a certain type of nanofluid known as “carbon nanotubes”. The water-based magnetohydrodynamic flow with the nanoparticles MWCNT and SWCNT over an axially rotating stretching disk is highlighted in this article. In addition, the perspectives of viscous dissipation and MHD were taken into consideration. In order to formulate the physical problem, Xue’s model is considered with the thermophysical properties and characteristics of carbon nanofluid. The current modeled system of partial differential equations is transformed into an ordinary differential equation system by the suggesting of the best similarity technique. Later, the transformed system of ordinary differential equations is solved numerically by using the Keller box method and the shooting method. Figures and charts are used to study and elaborate the physical behavior of the key subjective flow field parameters. The saturation in the base fluid is considered in both kinds of carbon nanotubes, the single-wall (SWCNTs) and the multiwall (MWCNTs). It is noted that the heat transfer mechanism shows some delaying behavior due to the increase in the Eckert number and the volume fraction elevation values. For the larger volume fraction values and the magnetic parameter, the skin friction increases. In addition, while the temperature profile increases with the Biot numbers, it falls for the increasing values of the Prandtl number. Furthermore, it is noted that the irreversibility of the thermal energy is influenced by the Biot number, temperature difference, Brinkmann number, and magnetic field, which all have dynamic effects on the entropy and the Bejan number.

## 1. Introduction

The nanofluid study is currently a leading scientific area because of its broad range of activities in oils, water, solar power, and mechatronics. Chemotherapy with nanoparticles is used to kill infected censorial cells. Nano-liquids offer enhanced thermophysical properties, such as thermal diffusivity and conductivity, and are crucial in many industrial applications, such as shipbuilding, nuclear power stations, thermosyphons, pulsing heat pipes, and biotechnology. Nanomaterials are innovative because they are potentially helpful in various mass transportation systems, heat transportation applications such as cooling machines, pharmaceuticals, nuclear reactors, electronics, solar collectors, fuel, and residential cooling processes. Although metal has a higher thermal conductivity rate in solids than in water and oil, its thermal conductivity can be improved by dispersing metallic nanoparticles in base fluids. This notion was also postulated by Choi [1] as a mix of basic fluids and colloidal nanoparticles and is called nanofluids. The physical properties of nano-liquids change dramatically as the temperature and viscosity are increased. Nanofluids have been developed to produce thermal fluids. Ayodeji et al. [2] studied the flow of nanofluid MHD across a stretching surface with dissipation effects and slip. Khan et al. [3] investigated the magnetic properties of Newtonian fluids as a result of the transition of paraboloid and chemically bonded bioconvection species. Khan took into consideration the phenomena of fluid flow with cylinder-shaped CNTs on a flat plate with heat transfer and the Navier slip [4]. In order to examine the flow of nanotubes in the rotating medium, Seth [5] chose porous media. The results were derived by the flow phenomena for heat dissipation and Joule heating. Such flows had a magnificent effect on industrial and mechanical processes. Hayat et al. [6] evaluated the fluid flow of the CNTs to a stagnation point on a nonlinear stretching sheet with uniform thickness. This caused both homogeneous and heterogeneous reactions in the melting of the heat and chemical reactions of an autocatalyst. It was ultimately concluded that multiwall nanotubes of carbon were accelerating the flow at higher speeds than the single-wall nanotubes. In an investigation of a spinning stretching channel exhibiting radiative heat flux, the flow of CNTs was also investigated by Ghadikolaei et al. [7]. Many researchers [8,9,10,11,12,13] have demonstrated numerically the magnifying of the heat conductivity in different aspects after Eastman [14] and Choi [15].

The fluid flow as a result of a rotating disk has attracted a lot of attention; the reason for this is that it covers a wide range of geophysical applications, including Earth rotation, fluid movement in the Earth’s mantle near the crust, surface rotation, rotors, and reactors. Von Karman [16] was the first to explain the impact of fluid flow on a rotating disk. Following Karman’s influential work, eminent researchers have addressed these transformations in a variety of physical situations. The flow is required in the expulsion of polymers, metals, paper, and fiber glass, among other things, due to the stretched surface. Heat transfer and the stretching rate have a huge impact on the output product quality. Heat transfer causes heated metals to cool to a specific temperature. It is ensured that the metals do not approach the melting point in a heat process. These processes are carried out in order to make metals highly resistant. The three-dimensional flow caused by a rotating, stretching surface was explored [17], and Wang introduced a parameter λ that denoted the comparison of rotation and stretching. Fang [18] mainly examined the steady flow on a rotating stretching disk. On a rotating disk that stretches radially at the same time, three-dimensional steady flow was also investigated. Weidman [19] envisioned a flow of axisymmetric stagnation points affecting a rotating disk while radially stretching. Weidman investigated two types of stagnation flows: the Homann stagnation flow and the rotating Agarwal stagnation flow.

Entropy is the amount of inaccessible energy in a closed thermodynamic system. Quality and energy are very important parameters for the design and development of engineering products. The second law of thermodynamics gives us a way to determine the quality and extent of energy degradation. Irreversibility or entropy is the important approach to determining energy quality. In accordance with the second thermodynamics law, transforming energy into useful work is an energy loss that reduces the efficiency of devices for energy conversion. The energy degradation is equivalent to the production of entropy. Therefore, entropy production in a system causes the amount of energy available due to the disordered behavior of the fluid. Through reduced entropy generation, the productivity of the thermal system can be enhanced technically. In order to reduce entropy production, it is therefore very important to know how the entropy production is distributed during the thermodynamic process. Entropy production minimization is necessary for the maximal utility of the flow problems and thermal systems [20,21,22,23,24,25,26,27,28]. The flow of spinning nanofluid with entropy generation incorporated in the thermal slip was studied by Rehman et al. [29]. Recent research has looked at the effects of various constraints on liquid flow and heat transmission in a spinning fluid across a stretched disk [30,31,32,33]. Biswas et al. [34,35] studied the several cases of nanofluid heat transfer in a W-shaped geometry. Ashraf et al. [36,37,38,39] investigated the transient mixed convection flow along different surfaces numerically.

In order to eliminate the energy wasted, scientists took drastic actions. They revised the energy conversion equipment and created products and methods to use the established resources better. The current study aims to obtain a numerical solution for a steady, MHD, and incompressible rotating flow of carbon nanotubes over a rotating stretching disk with a viscous dissipation effect using the shooting method and the Keller box scheme. The single-wall and double-wall carbon nanotubes in the water base are taken into consideration. The formulated problem is resolved, and the results are thoroughly investigated. Finally, it presents and analyzes the behaviors of the physical parameters on the temperature and velocity profiles. Furthermore, Xue’s model is used to formulate the problem. In accordance with this model, thermal conductivity is given by:

$$\frac{k_{nf}}{k_{f}}=\frac{1-\phi +2\phi \left(\frac{k_{CNT}}{k_{CNT}-k_{f}}\right)\ln\left(\frac{k_{CNT}+k_{f}}{2k_{f}}\right)}{1-\phi +2\phi \left(\frac{k_{f}}{k_{CNT}-k_{f}}\right)\ln\left(\frac{k_{CNT}+k_{f}}{2k_{f}}\right)}$$

## 2. Problem Statement

**Figure 1:**
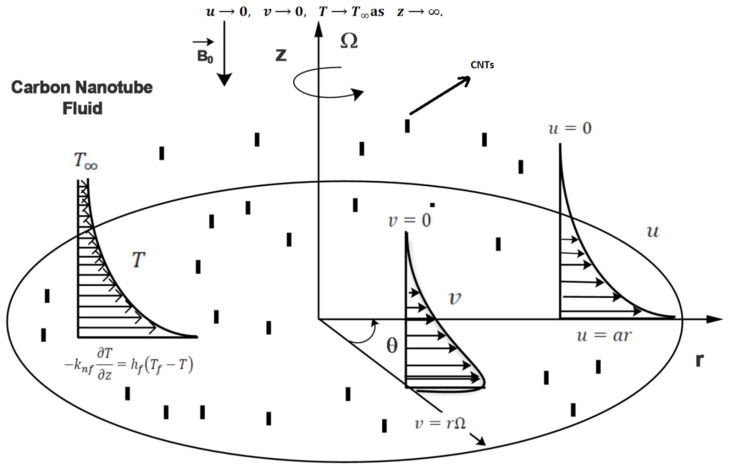
Geometry and coordinates of model.

The steady three-dimensional flow of an incompressible carbon nanofluid over a radially stretching and rotating disk is investigated. The disk is considered to be in a plane with z ≥ 0. Figure 1 illustrates the flow geometry as well as the coordinate system.

The flow is assumed to have a radial velocity u=ar and an azimuthal velocity v=Ωr, where a is the strain rate and Ω is the anticlockwise angular velocity of the disk. In the transverse direction of flow, the uniform magnetic field $B_{o}$ is produced. Because of the small Reynold number, the electric field is absent. It is also assumed that a heated fluid just below the disk is used to change the temperature of the disk via convective heat transfer, which yields the heat transfer coefficient $h_{f}$. Joule heating is considered. We have the following equations for the above flow by Weidman [19]:

The mass conservation equation:

(1) $$\frac{\partial u}{\partial r}+\frac{u}{r}+\frac{\partial w}{\partial z}=0,$$

The radial momentum equation:

(2) $$u\frac{\partial u}{\partial r}+w\frac{\partial u}{\partial z}-\frac{v^{2}}{r}=\nu_{nf}\left(\frac{\partial^{2}u}{\partial r^{2}}+\frac{1}{r}\frac{\partial u}{\partial r}-\frac{u}{r^{2}}+\frac{\partial^{2}u}{\partial z^{2}}\right)-\frac{\sigma_{nf}}{\rho_{nf}}B_{o}^{2}\;u,$$

The azimuthal momentum equation:

(3) $$u\frac{\partial v}{\partial r}+w\frac{\partial v}{\partial z}+\frac{uv}{r}=\nu_{nf}\left(\frac{\partial^{2}v}{\partial r^{2}}+\frac{1}{r}\frac{\partial v}{\partial r}-\frac{v}{r^{2}}+\frac{\partial^{2}v}{\partial z^{2}}\right)-\frac{\sigma_{nf}}{\rho_{nf}}B_{o}^{2}v,$$

The axial momentum equation:

(4) $$u\frac{\partial w}{\partial r}+w\frac{\partial w}{\partial z}=\nu_{nf}\left(\frac{\partial^{2}w}{\partial r^{2}}+\frac{1}{r}\frac{\partial w}{\partial r}+\frac{\partial^{2}w}{\partial z^{2}}\right),$$

The energy equation:

(5) $$u\frac{\partial T}{\partial r}+w\frac{\partial T}{\partial z}=\frac{K_{nf}}{(\rho c_{p)}{}_{nf}}\left(\frac{\partial^{2}T}{\partial r^{2}}+\frac{1}{r}\frac{\partial T}{\partial r}+\frac{\partial^{2}T}{\partial z^{2}}\right)+\frac{\sigma_{nf}B_{o}^{2}}{{\left(\rho c_{p}\right)}_{nf}}\left(u^{2}+v^{2}\right)$$

The boundary equations:

(6) $$u=ar,v=\Omega r,\;w=0,-k_{nf}\frac{\partial T}{\partial z}=h_{f}\left(T_{f}-T\right),\mathrm{at}\;z=0.$$

(7) $$\begin{array}{l} u\to 0,\;v\to 0,\;T\to T_{\infty }\mathrm{as}\;z\to \infty . \end{array}$$

where the radial, azimuthal, and axial components of velocity are (*u*, *v*, *w*), and $\nu_{nf}$, $\alpha_{nf}$, and ${\left(\rho c_{p}\right)}_{nf}$ are the efficient kinematic viscosity, thermal diffusion, and heat capacity of the nanofluid, respectively. $T_{f}$ is the temperature of the heated fluid, and $T_{\infty }$ is the ambient fluid temperature. Xue’s model is utilized for the thermal conductivity of the carbon nanofluid and is expressed in Equation (11).

The efficient properties of the nanofluid are expressed as:

(8) $$\rho_{nf}=\left(1-\phi \right)\rho_{f}+\phi \rho_{CNT},$$

(9) $$\mu_{nf}=\frac{\mu_{f}}{{\left(1-\phi \right)}^{2.5}},\;\begin{array}{l} \nu_{nf}=\frac{\mu_{nf}}{\rho_{nf}},\alpha_{nf}=\frac{k_{nf}}{{\left(\rho c_{p}\right)}_{nf}} \end{array}$$

(10) $${\left(\rho c_{p}\right)}_{nf}=\left(1-\phi \right){\left(\rho c_{p}\right)}_{f}+\phi {\left(\rho c_{p}\right)}_{CNT}$$

(11) $$\frac{k_{nf}}{k_{f}}=\frac{1-\phi +\phi \left(\frac{k_{CNT}}{k_{CNT-k_{f}}}\right)ln\left(\frac{k_{CNT+k_{f}}}{2k_{f}}\right)}{1-\phi +\phi \left(\frac{k_{f}}{k_{CNT-k_{f}}}\right)ln\left(\frac{k_{CNT+k_{f}}}{2k_{f}}\right)}$$

Here, $\rho_{nf}$ is the density, $\mu_{nf}$ is the dynamic viscosity, $k_{nf}$ is the thermal conductivity, and ϕ is the solid volume fraction of the nanofluid. $\rho_{CNT}$ is the density, and ${\left(\rho c_{p}\right)}_{CNT}$ is the heat capacity of the carbon nanotubes. $\rho_{f}$ is the density, $\mu_{f}$ is the dynamic viscosity, and ${\left(\rho c_{p}\right)}_{f}$ is the heat capacity of the base fluid. The following similarity transitions are used to obtain the non-linear ordinary differential equations.

(12) $$\begin{array}{l} u\left(r,z\right)=u_{w}f^{\prime }\left(\eta \right)=arf^{\prime }\left(\eta \right),\;v\left(r,\eta \right)=u_{w}g\left(\eta \right)=arg\left(\eta \right),\\ w\left(r,z\right)=-2\sqrt{a\nu_{f}}f\left(\eta \right),\;\theta \left(\eta \right)=\frac{T-T_{\infty }}{T_{f}-T_{\infty }},\;\eta =\sqrt{\frac{a}{\nu_{f}}}z \end{array}\}$$

Equation (1) is satisfied, and Equations (2)–(7) will take the form after applying Equation (12) as:

(13) $$f^{\prime \prime \prime }-M^{2}{\left(1-\phi \right)}^{2.5}f^{\prime }+(1-\phi +\phi \left(\rho {)}_{CNT}/\rho_{f}\right)(2ff^{\prime \prime }-{f^{\prime }}^{2}+g^{2})=0$$

(14) $$g^{\prime \prime }-M^{2}{\left(1-\phi \right)}^{2.5}g+(1-\phi +\phi \left(\rho {)}_{CNT}/\rho_{f}\right)\left(2fg^{\prime }-2f^{\prime }g\right)=0,$$

(15) $$\frac{k_{nf}}{k_{f}}\theta^{\prime \prime }+2\mathrm{Pr}(1-\phi +\phi {\left(\rho c_{p}\right)}_{CNT}/\left(\rho c_{p}{)}_{f}\right)f\theta^{\prime }+\mathrm{Pr}Ec\;M^{2}\;\left(u^{2}+v^{2}\right)=0,$$

(16) $$\begin{array}{l} f\left(0\right)=0,\;g\left(0\right)=S,f^{\prime }\left(0\right)=1,\theta^{\prime }\left(0\right)=-\frac{k_{f}}{k_{nf}}\sigma \left[1-\theta \left(0\right)\right] \end{array}$$

(17) $$\begin{array}{l} f^{\prime }\left(\infty \right)\to 0,\;g\left(\infty \right)\to 0,\theta \left(\infty \right)\to 0. \end{array}$$

The skin friction coefficient and Nusselt number for the fluid flow due to the rotating stretching disk are:

(18) $$C_{f}=\frac{1}{2\rho_{f}u_{w}^{2}}{\left[\tau_{r}^{2}+\tau_{\theta }^{2}\right]}^{1/2},\;Nu=\frac{r}{k_{f}\left[T_{f}-T_{\infty }\right]}{\left[-k_{nf}\frac{\partial T}{\partial z}|\right]}_{z=0}$$

Applying the similarity transformations, Equations (17) and (18) will take the form

(19) $$\frac{1}{2}Re^{\frac{1}{2}}C_{f}=\frac{1}{{\left(1-\phi \right)}^{2.5}}[{f^{\prime \prime }}^{2}\left(0\right)+{g^{\prime }}^{2}\left(0\right)]{}^{\frac{1}{2}},$$

(20) $$\frac{1}{2}Re^{-\frac{1}{2}}Nu=-\frac{k_{nf}}{k_{f}}\theta^{\prime }\left(0\right)$$

The non-dimensional parameters are the Reynold number, the magnetic field parameter, the Eckert number, the rotational parameter, the Biot number, and the Prandtl number, as follows:

(21) $$Re=\frac{au_{w}}{\nu_{f}},\;M^{2}=\frac{\sigma_{f}}{a{\left(\rho \right)}_{f}},\;Ec=\frac{u_{w}^{2}}{{\left(c_{p}\right)}_{f}\left(T_{f}-T_{\infty }\right)},\;S=\frac{\Omega }{a},\;\sigma =\frac{h_{f}}{k_{f}}\sqrt{\frac{\nu_{f}}{a}},\;\mathrm{Pr}=\alpha_{f}/\nu_{f}$$

## 3. Entropy Generation

To understand the irreversibility of a system’s thermal energy, it is necessary to investigate entropy generation. The rate at which entropy is produced per unit volume for the three-dimensional carbon nanotube flow on a stretched and rotating disk is provided in accordance with approximated boundary layers.

(22) $$E_{G}^{\prime \prime }=\frac{k_{nf}}{T^{2}{}_{\infty }}{\left(\Delta T\right)}^{2}+\frac{\mu_{nf}}{T_{\infty }^{2}}\Phi =\frac{k_{nf}}{T^{2}{}_{\infty }}{\left(\frac{\partial T}{\partial z}\right)}^{2}+\frac{\sigma_{nf}}{T_{\infty }}B_{o}^{2}\left(u^{2}+v^{2}\right)$$

The entropy related to heat transfer is represented by the term (1) on the right-hand side of the expression (22), while the entropy due to viscous dissipation is indicated by the second term. The entropy production characteristics are defined by the boundary conditions (14) and (15).

(23) $${\left(E_{G}^{\prime \prime }\right)}_{o}=\frac{k_{f}}{T_{\infty }^{2}r^{2}}\left(T_{f}-T_{\infty }\right)$$

The induced similarity variables define the dimensionless entropy production:

(24) $$N_{G}=\frac{E_{G}^{\prime \prime }}{{\left(E_{G}^{\prime \prime }\right)}_{o}}=\frac{k_{nf}}{k_{f}}Re\theta^{\prime }+\frac{H_{a}^{2}}{\Lambda }B_{r}\;\left(f^{\prime }{}^{2}+g^{2}\right)$$

where

(25) $$B_{r}=Pr.\;Ec=\frac{\mu_{f}u_{w}^{2}}{k_{f}\left(T_{f}-T_{\infty }\right)}\;(\mathrm{Brickman}\;\mathrm{number}),$$

$$\Lambda =\frac{\left(T_{f}-T_{\infty }\right)}{T_{\infty }}\;(\mathrm{Temperature}\;\mathrm{difference}\;\mathrm{parameter})$$

The distribution of the entropy generation in the flow domain is determined by the entropy generation number $\;N_{G}$. To solve the problem, the involvement of thermal conductivity in the entropy production compared to the total entropy production must be calculated. The Bejan number indicates the relevance of the thermal irreversibility in comparison to the overall irreversibility and is defined as

(26) $$B_{e}=\frac{\frac{k_{nf}}{k_{f}}Re\theta^{\prime^{2}}}{\frac{k_{nf}}{k_{f}}Re\theta^{\prime }+\frac{H_{a}^{2}}{\Lambda }B_{r}\;\left(f^{\prime }{}^{2}+g^{2}\right)}$$

## 4. Numerical Scheme

Two numerical schemes are used to solve the transformed problem described by Equations (13)–(17): the Keller box method (an implicit finite difference scheme) and the shooting method. The differential equations are modified to a system of the first order to enact both numerical methodologies. We explain each step further in detail.

### 4.1. Step 1

Firstly, all the differential equations are contracted to first-order equations.

(27) $$f^{\prime }=U,f^{\prime \prime }=V,\;g^{\prime }=P,\;\theta^{\prime }=Q,$$

(28) $$V^{\prime }-A_{1}U+A_{2}fV-A_{2}U^{2}+A_{2}g^{2}=0,$$

(29) $$P^{\prime }-A_{1}g+A_{2}Pf-A_{2}gU=0$$

(30) $$A_{3}Q^{\prime }+A_{4}fQ+A_{5}U^{2}+A_{5}V^{2}=0$$

(31) $$\begin{array}{c} A_{1}=M^{2}\left(1-\phi {)}^{-2.5}\right],\;A_{2}=2Pr(1-\phi +\phi \left(\rho {)}_{CNT}/\rho_{f}\right),\\ A_{3}=\frac{k_{nf}}{k_{f}},\;A_{5}=PrEcM^{2},\;A_{4}=EcPr/[(1-\phi +\phi {\left(\rho c_{p}\right)}_{CNT}/{\left(\rho c_{p}\right)}_{f} \end{array}\}$$

The boundary conditions are modified as:

(32) $$\begin{array}{l} f\left(0\right)=0,\;U\left(0\right)=1,\;Q\left(0\right)=-\frac{k_{f}}{k_{nf}}\sigma \left[1-\theta \left(0\right)\right]\\ g\left(0\right)=S,\;U\left(\infty \right)=0,\;g\left(\infty \right)=0,\;\theta \left(\infty \right)=0\}. \end{array}$$

### 4.2. Step 2

The following important points are explored to discretize Equations (27)–(30).

(33) $$\eta_{0}=0,\;\eta_{j}=\eta_{j-1}+h_{j-1},\;j=1,2,3,\ldots ,J,\;\eta_{J}=\eta_{\infty }$$

We differentiated Equations (27)–(30) at $\eta_{j-\frac{1}{2}}$ in the following way.

(34) $$h_{j-1}^{-1}\left(f_{j}-f_{j-1}\right)=U_{j-\frac{1}{2}},$$

(35) $$h_{j-1}^{-1}\left(U_{j}-U_{j-1}\right)=V_{j-\frac{1}{2}},$$

(36) $$h_{j-1}^{-1}\left(V_{j}-V_{j-1}\right)-A_{1}U_{j-\frac{1}{2}}+A_{2}{\left(fV\right)}_{j-\frac{1}{2}}-A_{2}{\left(U^{2}\right)}_{j-\frac{1}{2}}+A_{2}{\left(g^{2}\right)}_{j-\frac{1}{2}}=0,$$

(37) $$h_{j-1}^{-1}\left(g_{j}-g_{j-1}\right)=P_{j-\frac{1}{2}},$$

(38) $$h_{j-1}^{-1}\left(P_{j}-P_{j-1}\right)-A_{1}g_{j-\frac{1}{2}}+A_{2}{\left(Pf\right)}_{j-\frac{1}{2}}-A_{2}{\left(Ug\right)}_{j-\frac{1}{2}}=0,$$

(39) $$h_{j-1}^{-1}\left(\theta_{j}-\theta_{j-1}\right)=Q_{j-\frac{1}{2}},$$

(40) $$A_{3}h_{j-1}^{-1}\left(Q_{j}-Q_{j-1}\right)+A_{4}{\left(fQ\right)}_{j-\frac{1}{2}}+A_{5}{\left(V^{2}\right)}_{j-\frac{1}{2}}+A_{5}{\left(U^{2}\right)}_{j-\frac{1}{2}}=0,$$

(41) $$\begin{array}{l} f_{o}=0,U_{o}=1,g_{o}=S,Q_{o}=-\frac{k_{f}}{k_{nf}}\sigma \left[1-\theta \left(0\right)\right],U_{J}=0,g_{J}=0,\theta_{J}=0, \end{array}$$

### 4.3. Step 3

As shown in the Equations (34)–(40), the remodelled system of algebraic equations is non-linear in nature. Now we deploy Newton’s approach to linearize these equations.

(42) $$\begin{array}{l} f_{j}=f_{j}+\delta f_{j},U_{j}=U_{j}+\delta U_{j},V_{j}=V_{j}+\delta V_{j},\\ g_{j}=g_{j}+\delta g_{j},P_{j}=P_{j}+\delta P_{j},\theta_{j}=\theta_{j}+\delta \theta_{j}, \end{array}$$

By swapping expressions (42) in Equations (34)–(40) and ignoring the second and higher order of δ, we obtain the result shown in step 4.

### 4.4. Step 4

As a result, in order to solve the linear equations, the entire system is expressed in a matrix form using the block tridiagonal approach.

(43) $$\delta f_{j}-\delta f_{j-1}-\frac{1}{2}h_{j-1}\left(\delta U_{j}+\delta U_{j-1}\right)={\left({ϒ}_{1}\right)}_{j-\frac{1}{2}},$$

(44) $$\delta U_{j}-\delta U_{j-1}-\frac{1}{2}h_{j-1}\left(\delta V_{j}+\delta V_{j-1}\right)={\left({ϒ}_{2}\right)}_{j-\frac{1}{2}},$$

$${\left(\zeta_{1}\right)}_{j}\delta V_{j}+{\left(\zeta_{2}\right)}_{j}\delta V_{j-1}+{\left(\zeta_{3}\right)}_{j}\delta f_{j}+{\left(\zeta_{4}\right)}_{j}\delta f_{j-1}+{\left(\zeta_{5}\right)}_{j}\delta U_{j}+{\left(\zeta_{6}\right)}_{j}\delta U_{j-1}$$

(45) $$+{\left(\zeta_{7}\right)}_{j}\delta g_{j}+{\left(\zeta_{8}\right)}_{j}\delta g_{j-1}={\left({ϒ}_{3}\right)}_{j-\frac{1}{2}},$$

(46) $$\delta g_{j}-\delta g_{j-1}-\frac{1}{2}h_{j-1}\left(\delta P_{j}+\delta P_{j-1}\right)={\left({ϒ}_{4}\right)}_{j-\frac{1}{2}},$$

$${\left(\zeta_{9}\right)}_{j}\zeta P_{j}+{\left(\zeta_{10}\right)}_{j}\zeta P_{j-1}+{\left(\zeta_{11}\right)}_{j}\delta f_{j}+{\left(\zeta_{12}\right)}_{j}\delta f_{j-1}+{\left(\zeta_{13}\right)}_{j}\delta g_{j}+{\left(\zeta_{14}\right)}_{j}\delta g_{j-1}$$

(47) $$+{\left(\zeta_{15}\right)}_{j}\delta U_{j}+{\left(\zeta_{16}\right)}_{j}\delta U_{j-1}={\left({ϒ}_{5}\right)}_{j-\frac{1}{2}},$$

(48) $$\delta \theta_{j}-\delta \theta_{j-1}-\frac{1}{2}h_{j-1}\left(\delta Q_{j}+\delta Q_{j-1}\right)={\left({ϒ}_{6}\right)}_{j-\frac{1}{2}},$$

$${\left(\zeta_{17}\right)}_{j}\delta Q_{j}+{\left(\zeta_{18}\right)}_{j}\delta Q_{j-1}+{\left(\zeta_{19}\right)}_{j}\delta f_{j}+{\left(\zeta_{20}\right)}_{j}\delta f_{j-1}+{\left(\zeta_{21}\right)}_{j}\delta V_{j}+{\left(\zeta_{22}\right)}_{j}\delta V_{j-1}$$

(49) $$+{\left(\zeta_{23}\right)}_{j}\delta Q_{j}+{\left(\zeta_{24}\right)}_{j}\delta Q_{j-1}={\left({ϒ}_{7}\right)}_{j-\frac{1}{2}},$$

The boundary conditions are

(50) $$\delta f_{o}=0,\;\delta g_{o}=0,\;\delta U_{o}=0,\;\delta Q_{o}=0,U_{J}=0,\;\delta g_{J}=0,\;\delta \theta_{J}=0,$$

also

(51) $${\left({ϒ}_{1}\right)}_{j-\frac{1}{2}}=f_{j-1}-f_{j}+h_{j-1}U_{j-\frac{1}{2}},$$

(52) $${\left({ϒ}_{2}\right)}_{j-\frac{1}{2}}=U_{j-1}-U_{j}+h_{j-1}V_{j-\frac{1}{2}},$$

(53) $${\left({ϒ}_{3}\right)}_{j-\frac{1}{2}}=-[h_{j-1}^{-1}\left(V_{j}-V_{j-1}\right)-A_{1}U_{j-\frac{1}{2}}+A_{2}{\left(fV\right)}_{j-\frac{1}{2}}-A_{2}{\left(U^{2}\right)}_{j-\frac{1}{2}}+A_{2}\left(g^{2}{)}_{j-\frac{1}{2}}\right],$$

(54) $${\left({ϒ}_{4}\right)}_{j-\frac{1}{2}}=g_{j-1}-g_{j}+h_{j-1}P_{j-\frac{1}{2}},$$

(55) $${\left({ϒ}_{5}\right)}_{j-\frac{1}{2}}=-[h_{j-1}^{-1}\left(P_{j}-P_{j-1}\right)-A_{1}g_{j-\frac{1}{2}}+A_{2}{\left(fP\right)}_{j-\frac{1}{2}}-A_{2}\left(Ug{)}_{j-\frac{1}{2}}\right],$$

(56) $${\left({ϒ}_{6}\right)}_{j-\frac{1}{2}}=\theta_{j-1}-\theta_{j}+h_{j-1}Q_{j-\frac{1}{2}},$$

(57) $${\left({ϒ}_{7}\right)}_{j-\frac{1}{2}}=-[A_{3}h_{j-1}^{-1}\left(Q_{j}-Q_{j-1}\right)+A_{4}{\left(fQ\right)}_{j-\frac{1}{2}}+A_{5}{\left(V^{2}\right)}_{j-\frac{1}{2}}+A_{5}\left(U^{2}{)}_{j-\frac{1}{2}}\right],$$

The coefficients are



${\left(\zeta_{1}\right)}_{j}=h_{j-1}^{-1}+A_{2}f_{j},$



${\left(\zeta_{13}\right)}_{j}=-0.5A_{2}-0.5\;A_{2}U_{j},$



${\left(\zeta_{2}\right)}_{j}=-h_{j-1}^{-1}+A_{2}f_{j-1},$



${\left(\zeta_{14}\right)}_{j}=-0.5A_{2}-0.5\;A_{2}U_{j-1},$



${\left(\zeta_{3}\right)}_{j}=0.5A_{2}V_{j},$



${\left(\zeta_{15}\right)}_{j}=-0.5\;A_{2}g_{j},$



${\left(\zeta_{4}\right)}_{j}=0.5A_{2}V_{j-1},$



${\left(\zeta_{16}\right)}_{j}=-0.5\;A_{2}g_{j-1},$



${\left(\zeta_{5}\right)}_{j}=-0.5A_{1}+A_{2}U_{j},$



${\left(\zeta_{17}\right)}_{j}=A_{3}h_{j-1}^{-1}+0.5\;A_{4}f_{j},$



${\left(\zeta_{6}\right)}_{j}=-0.5A_{1}-A_{2}U_{j-1},$



${\left(\zeta_{18}\right)}_{j}=-A_{3}h_{j-1}^{-1}+0.5\;A_{4}f_{j-1},$



${\left(\zeta_{7}\right)}_{j}=-A_{2}g_{j},$



${\left(\zeta_{19}\right)}_{j}=0.5\;A_{4}Q_{j},$



${\left(\zeta_{8}\right)}_{j}=-A_{2}g_{j-1},$



${\left(\zeta_{20}\right)}_{j}=0.5\;A_{4}q_{j-1},$



${\left(\zeta_{9}\right)}_{j}=h_{j-1}^{-1}+0.5\;A_{2}f_{j},$



${\left(\zeta_{21}\right)}_{j}=A_{5}V_{j},$



${\left(\zeta_{10}\right)}_{j}=-h_{j-1}^{-1}+0.5\;A_{2}f_{j-1},$



${\left(\zeta_{22}\right)}_{j}=A_{5}V_{j-1},$



${\left(\zeta_{11}\right)}_{j}=0.5\;A_{2}P_{j},$



${\left(\zeta_{23}\right)}_{j}=A_{5}U_{j-1},$



${\left(\zeta_{12}\right)}_{j}=0.5\;A_{2}P_{j-1},$



${\left(\zeta_{24}\right)}_{j}=A_{5}U_{j-1}$



Equations (43)–(49) are a linear system of algebraic equations. To improve the solution, the system of equations is sought iteratively. The iterative procedure is stopped when the specified tolerance is obtained.

## 5. Numerical Results and Discussion

In the presence of viscous and magnetic effects, the second laws and the thermal transfer analysis of the CNT water-based nanofluid are managed to perform the theoretical behavior of above-mentioned mechanism. The Keller box scheme is used to solve the dimensionless set of nonlinear differential equations numerically. The nonlinear differential equations are also numerically solved by a shooting method for the validation of our numerical code. The appropriate estimations that satisfy all the boundary conditions are chosen to solve Equations (12)–(16) and to obtain a more accurate approximation of the solution. Table 1 shows the thermal properties of the nanofluids and water.

Table 2 includes the impact of the magnetic and rotation parameters on $f^{\prime }\left(0\right)$, $-g^{\prime }\left(0\right)$, and $-\theta^{\prime }\left(0\right)$ at two chosen magnetic parameters when viscous dissipation and Joule heating are not present. According to the previously shown figures, the larger the rotation, the larger all the physical quantities, together with the heat transfer. When these values are compared to those of Mustafa [36], it is revealed that the calculated results have excellent promise.

### 5.1. Velocity Profiles

**Figure 2:**
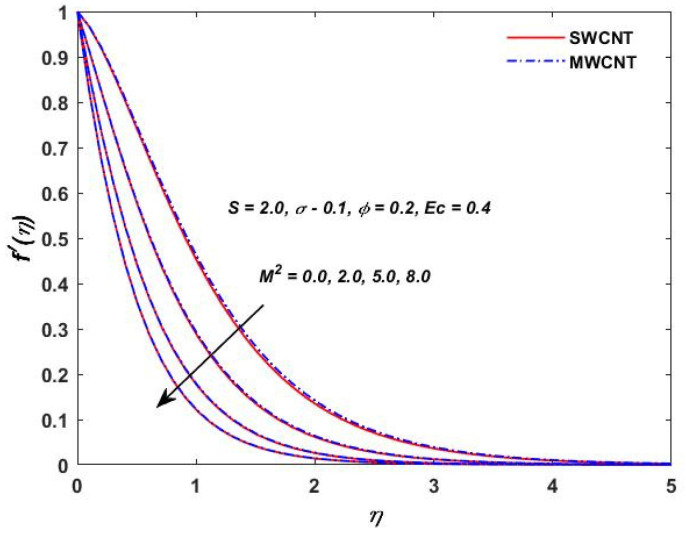
Magnetic parameter verses velocity $f^{\prime }\left(\eta \right)$.

**Figure 3:**
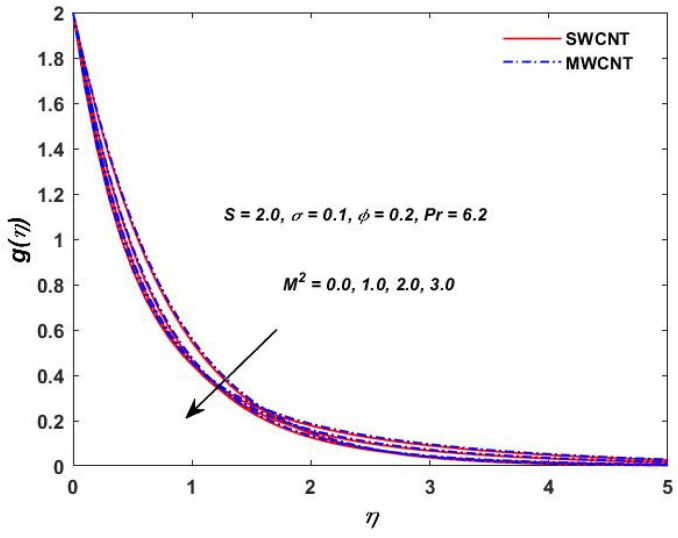
Magnetic parameter verses velocity g(η).

**Figure 4:**
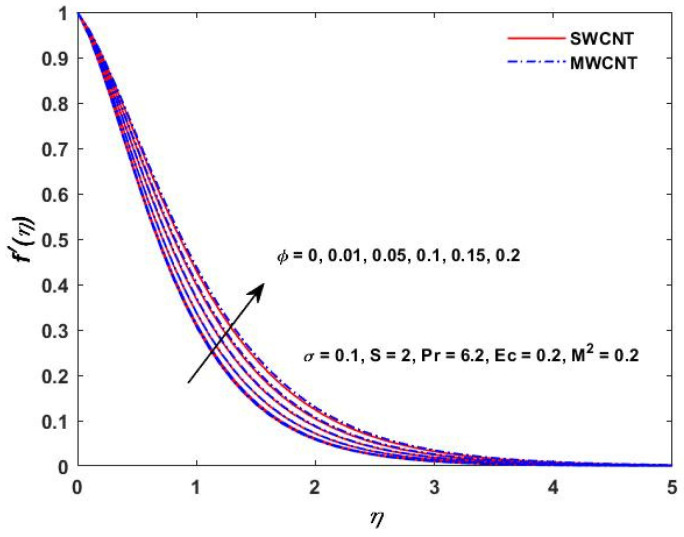
Volume fraction verses velocity $f^{\prime }\left(\eta \right)$.

**Figure 5:**
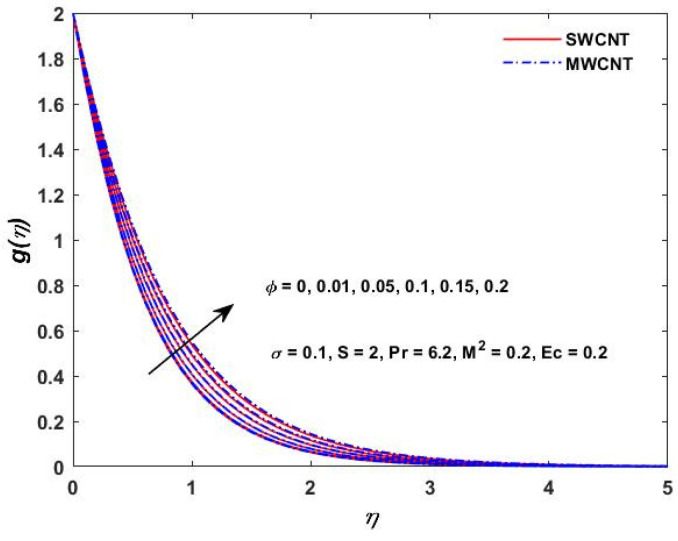
Volume fraction verses velocity g(η).

**Figure 6:**
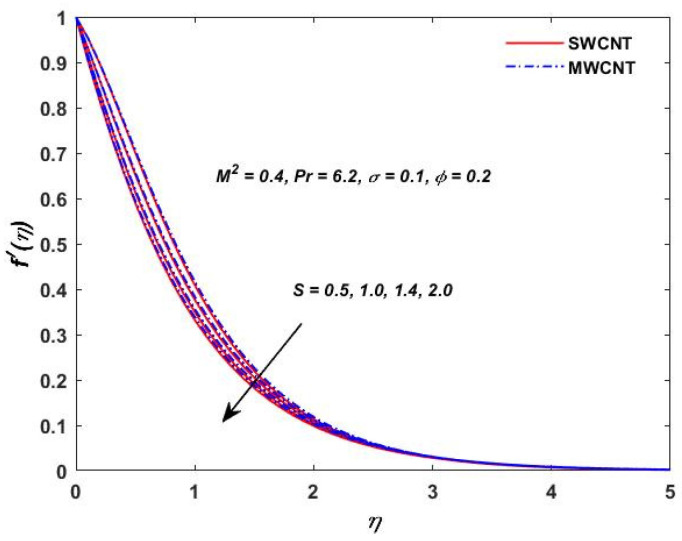
Rotation S verses velocity $f^{\prime }\left(\eta \right)$.

**Figure 7:**
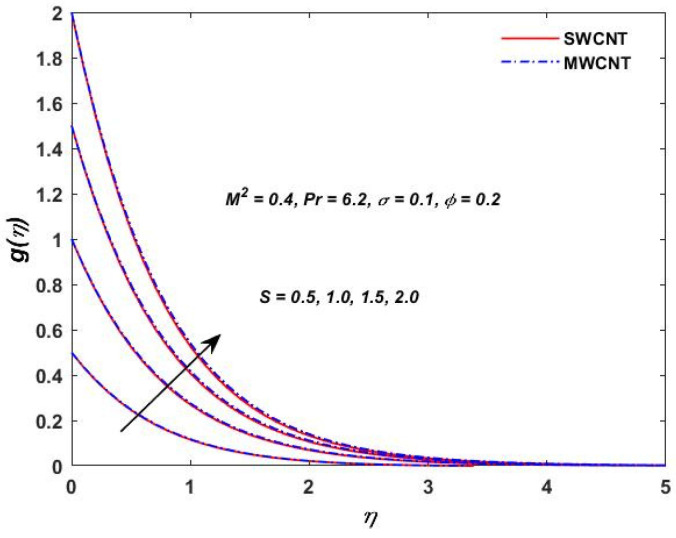
Rotation S verses velocity g(η).

The current study investigated the effect of the parameter ($M^{2}$) on the velocities $f^{\prime }\left(\eta \right)$ and g(η) profiles and are described in Figure 2 and Figure 3. The magnetic field parameter has a negative impact on the $f^{\prime }\left(\eta \right)$ and g(η) profiles according to the theory of the Lorentz force, i.e., the increments in the magnetic field parameter which reduce the $f^{\prime }\left(\eta \right)$ and g(η) profiles can be seen in the above-mentioned figures. The behavior of the velocities under volume fraction ϕ and the rotation S influences is indicated in Figure 4, Figure 5, Figure 6 and Figure 7. As shown in Figure 4 and Figure 5, an increasing solid volume value increases both velocities $f^{\prime }\left(\eta \right)$ and g(η). Figure 6 and Figure 7 show the effect of rotation parameter ‘S’ on the velocity profiles. Figure 7 depicts the increasing behavior as the rotation parameter is increased, whereas Figure 6 depicts the reverse effect.

### 5.2. Temperature Profiles

**Figure 8:**
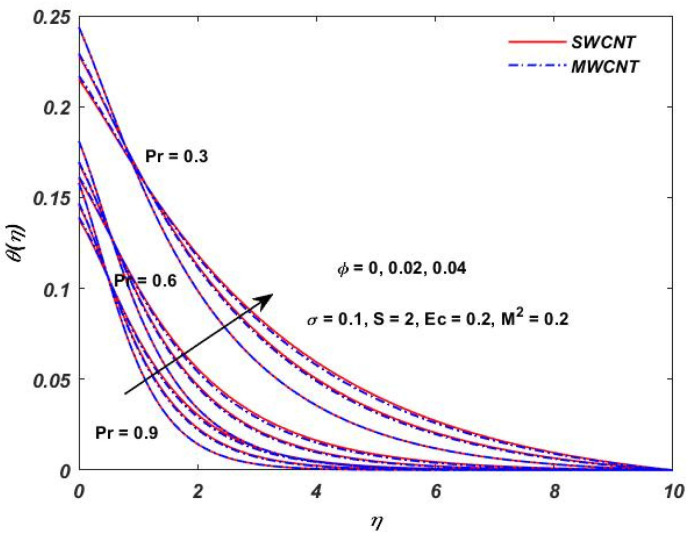
Prandtl (Pr) verses θ(η).

**Figure 9:**
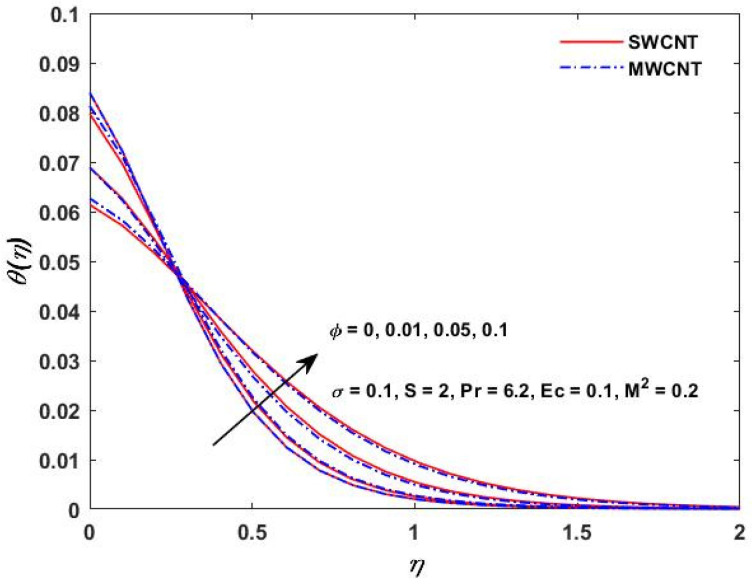
Solid volume ϕ verses θ(η).

**Figure 10:**
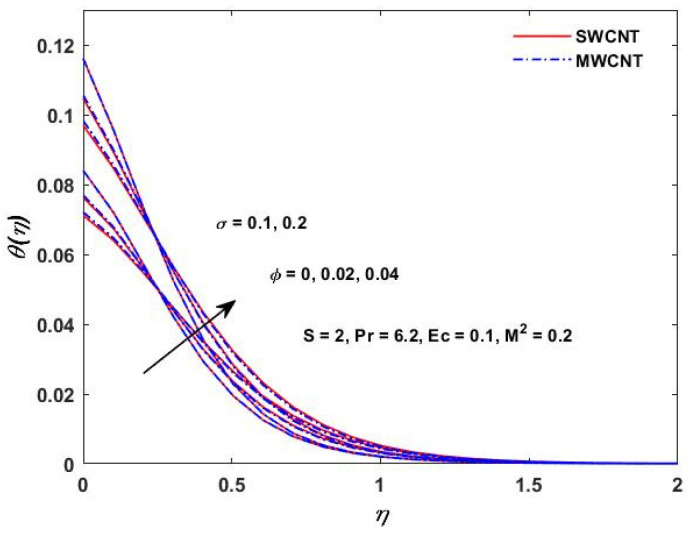
σ verses θ(η).

**Figure 11:**
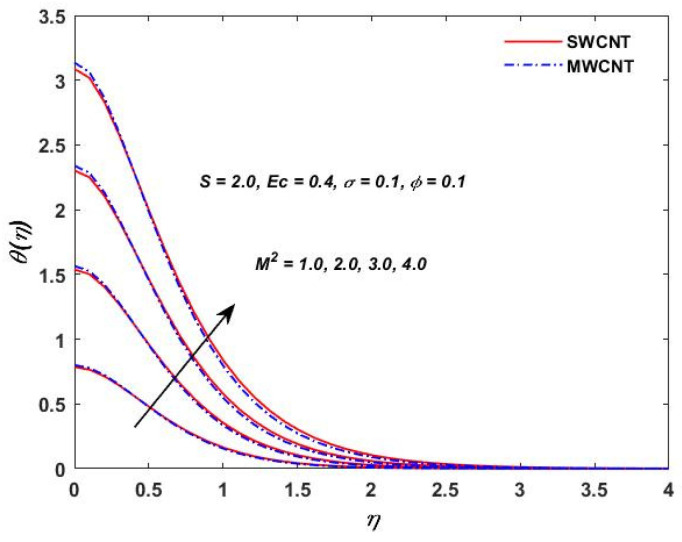
$M^{2}$ verses θ(η).

**Figure 12:**
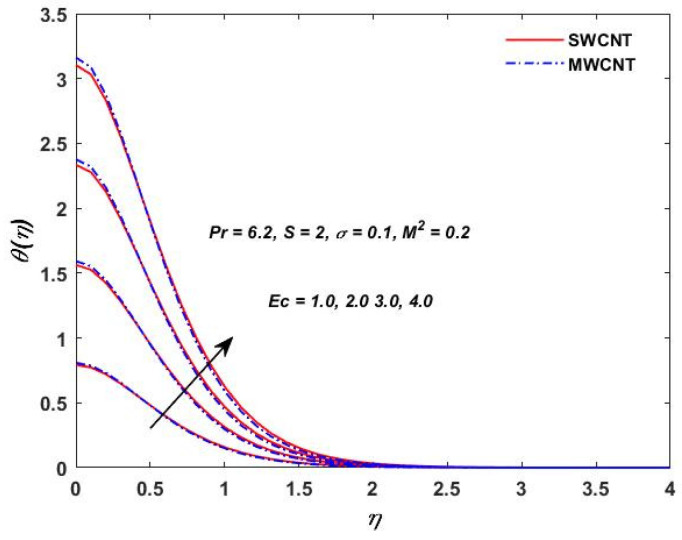
Eckert verses θη.

**Figure 13:**
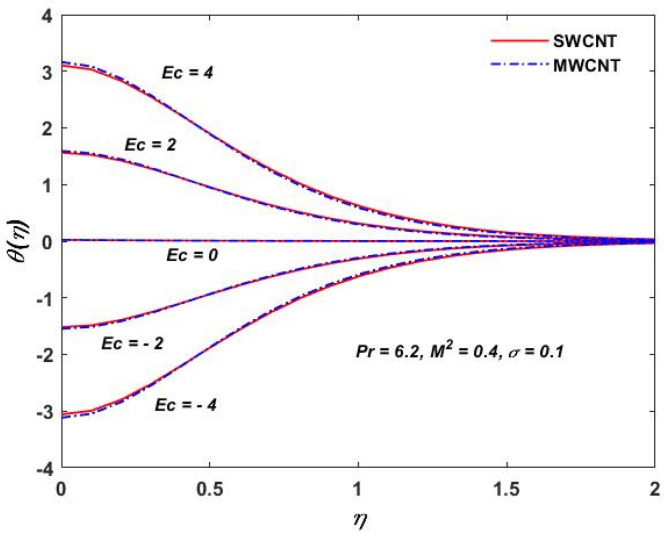
Eckert verses θ(η).

**Figure 14:**
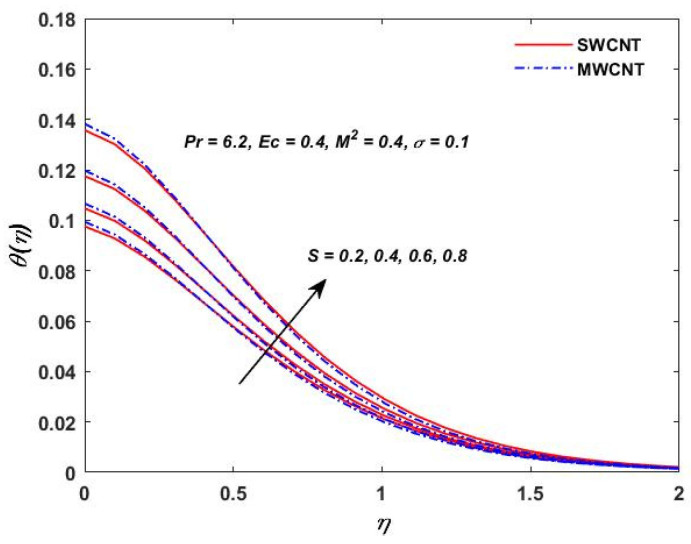
Rotation verses θ(η).

The influence of (Pr) on the temperature profile θ(η) is portrayed in Figure 8. Physically, nanofluids have a high thermal diffusivity while having a low (Pr), and vice versa. As a result, the liquid temperature drops. From here, we can see that the rising (Pr) has a decrease in θ(η). Figure 9 describes the temperature distribution impact of the volume fraction of the nanoparticles for both the SWCNTs and the MWCNTs. Higher ϕ results can be observed in both CNTs and in an improved thermal boundary layer thickness at a stronger temperature field. The influence of the Biot number on both CNTs is seen in Figure 10; an increase in the temperature is noticed when the amount of the Biot number is increased. The Biot number is well defined as the thermal resistance ratio of a solid to the thermal resistance of the boundary layer. Greater Biot numbers imply better convection and thicker thermal layers, resulting in more even temperature distribution in both types of CNTs. The effects of the magnetic parameter and the Eckert number are seen in Figure 11, Figure 12 and Figure 13. Figure 11 shows that temperature increases with the increasing magnetic values. The reason for this is that with the increasing magnetic values the ohmic heating grows, and thus, the fluid temperature is increased. The Eckert number is a measurement of the friction force in between fluid layers. Thus, the frictional heating increases by increasing the Eckert numbers, and this results in an increase in the fluid temperatures, as shown in Figure 12 and Figure 13. The temperature increases with the increasing rotating parameter when there is viscous dissipation and a magnetic parameter, as shown in Figure 14.

### 5.3. Variations of Skin Friction and Nusselt Number

**Figure 15:**
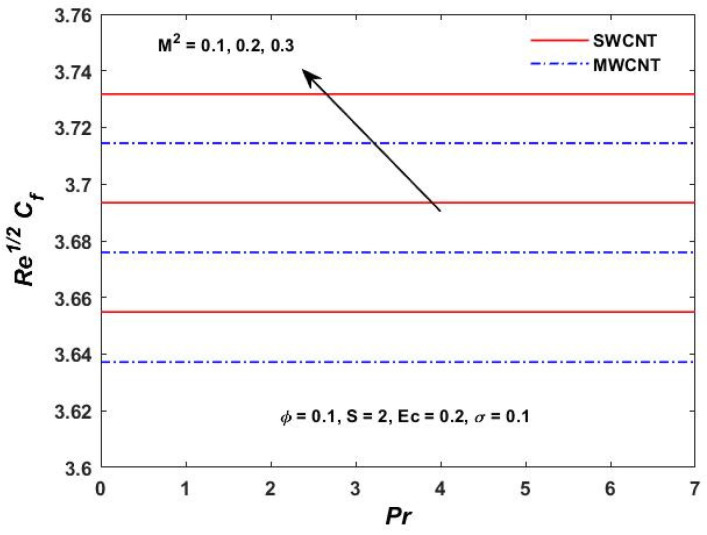
$M^{2}$ verses Prandtl and $Re^{1/2}C_{f}$.

**Figure 16:**
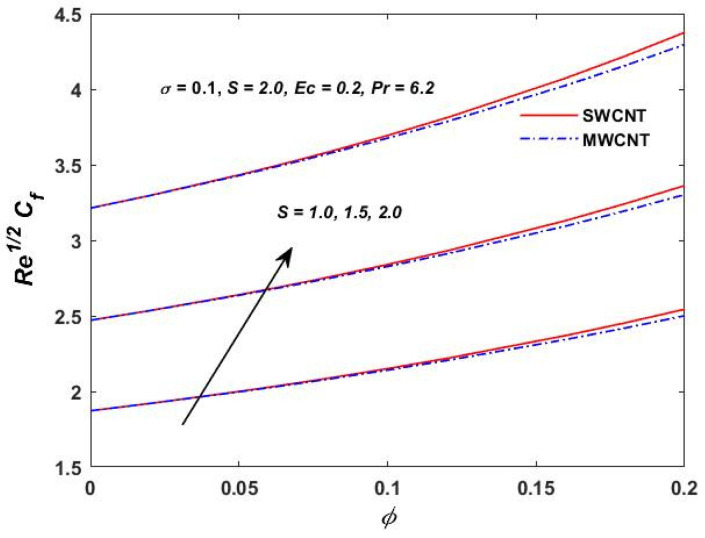
S verses $Re^{1/2}C_{f}$.

**Figure 17:**
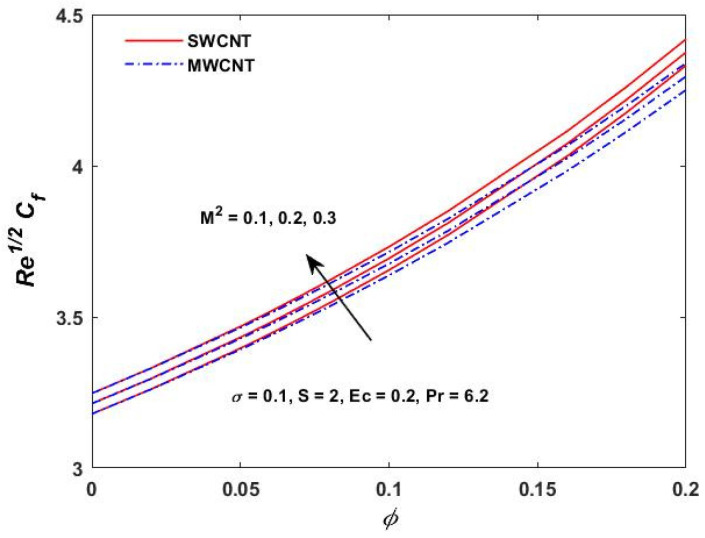
$M^{2}$ verses $Re^{1/2}C_{f}$.

**Figure 18:**
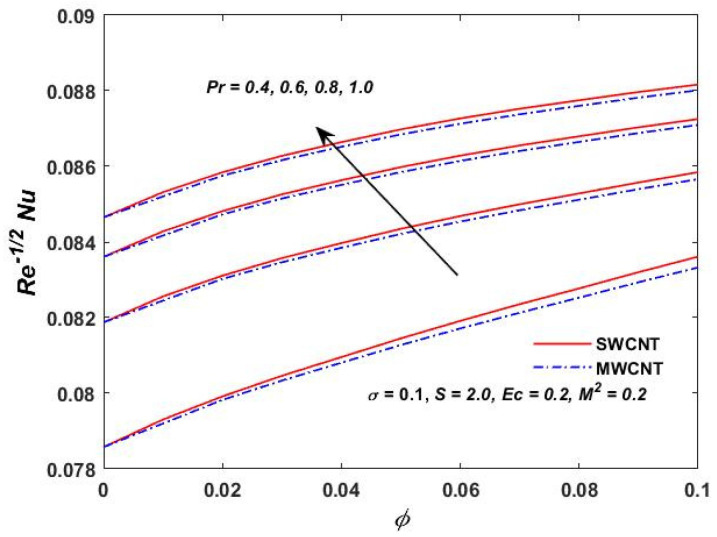
Prandtl verses $Re^{-1/2}Nu$.

**Figure 19:**
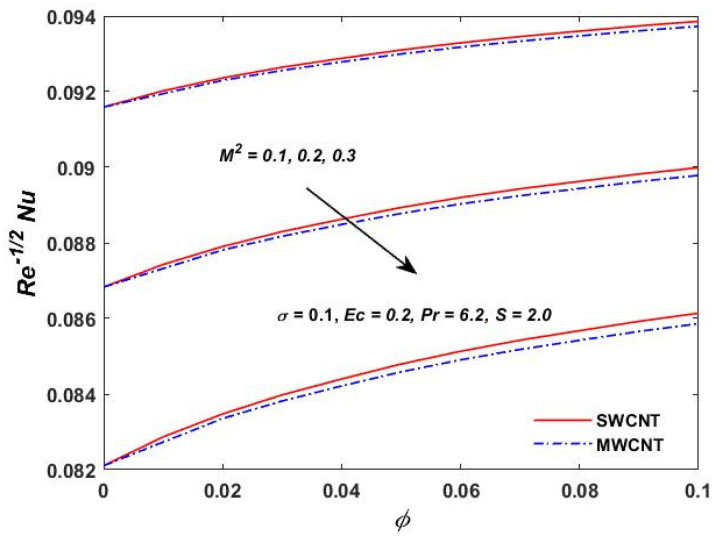
$M^{2}$ verses $Re^{-1/2}Nu$.

**Figure 20:**
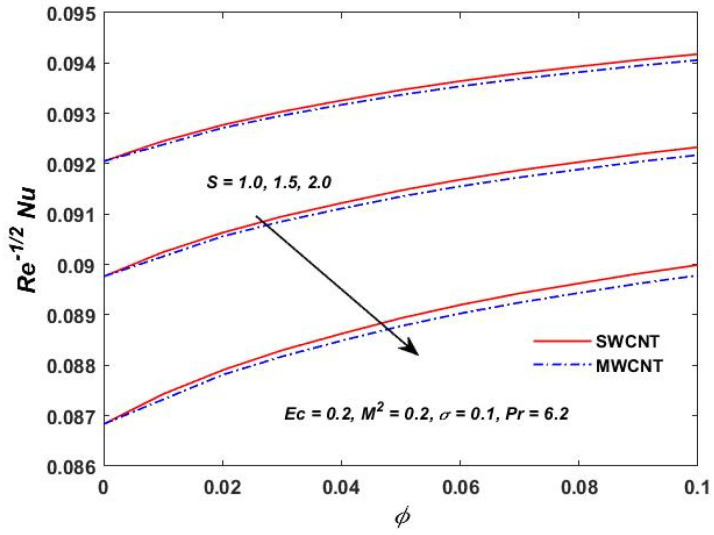
Rotation verses $Re^{-1/2}Nu$.

**Figure 21:**
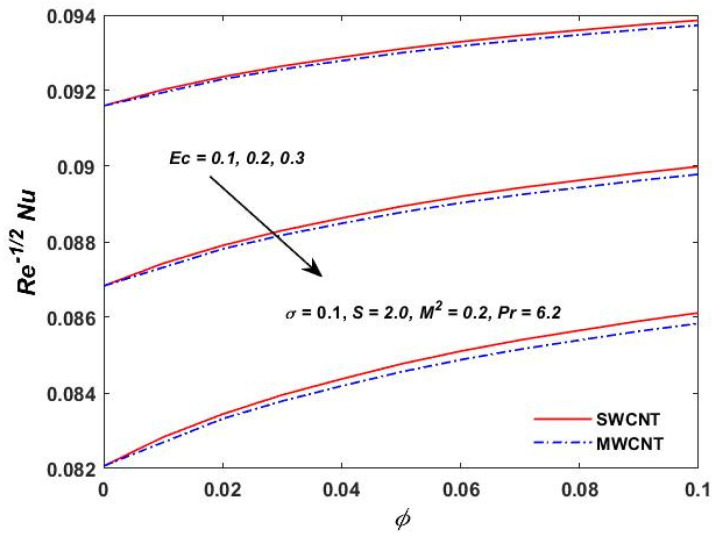
Eckert verses $Re^{-1/2}Nu$.

Figure 15, Figure 16 and Figure 17 show the variation of the skin friction as a function of the rotating parameter S and the magnetic parameter $M^{2}$. The skin friction has been observed to increase for both S and $M^{2}$ in both SWCNTs and MWCNTs. Figure 18, Figure 19, Figure 20 and Figure 21 show the Nusselt number behavior for the number of relevant parameters for both CNT types. According to Figure 18, the Nusselt number appears to increase for (Pr) in both CNTs. When the value of (Pr) is increased, the coefficient of heat transfer increases rapidly. These observations clearly show that the Prandtl number and the Nusselt number are directly proportional to each other. When looking at Figure 19, Figure 20 and Figure 21, it has been determined that the Nusselt number is a decreasing function of the magnetic parameters $M^{2}$ and Ec, whereas the increasing behavior is shown against rotation S, implying that as these parameters increase the rate of heat transfer declines.

### 5.4. Effects of Parameters on Entropy and Bejan Number

**Figure 22:**
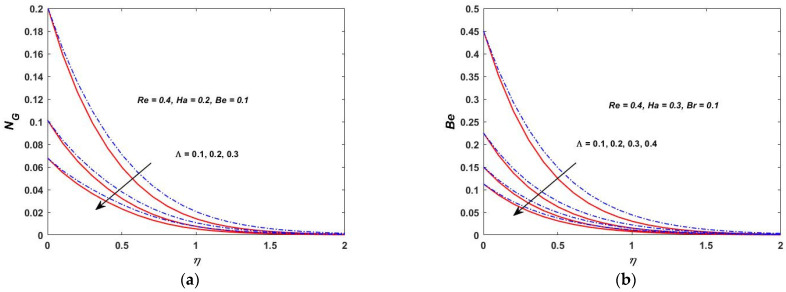
(**a**) Λ verses entropy $N_{G}$. (**b**) Λ verses Bejan (Be).

**Figure 23:**
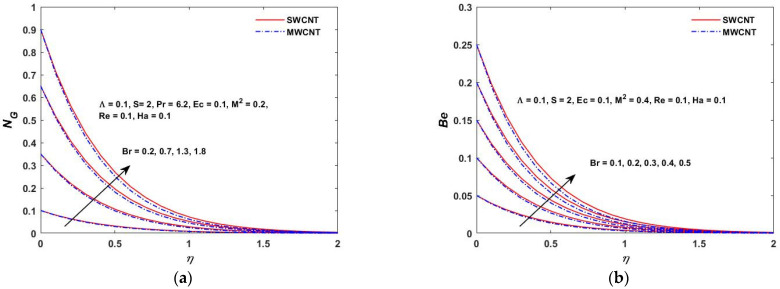
(**a**) Brickman verses entropy $N_{G}$. (**b**) Brickman verses Bejan (Be).

**Figure 24:**
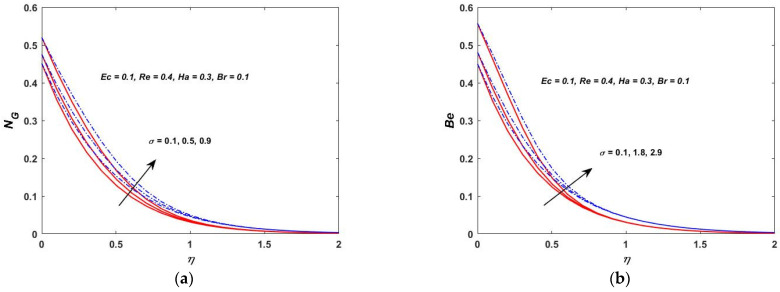
(**a**) Biot verses entropy $N_{G}$. (**b**) Biot verses Bejan (Be).

**Figure 25:**
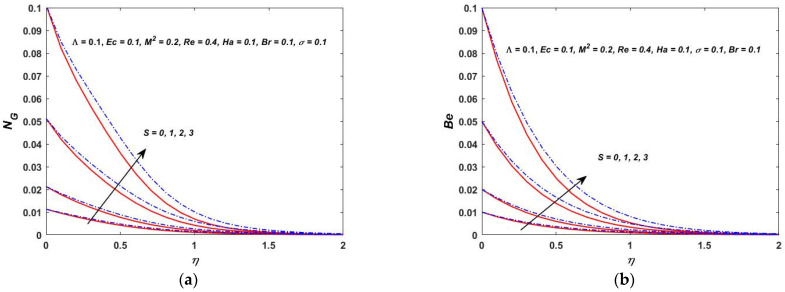
(**a**) Rotation verses entropy $N_{G}$. (**b**) Rotation verses Bejan (Be).

**Figure 26:**
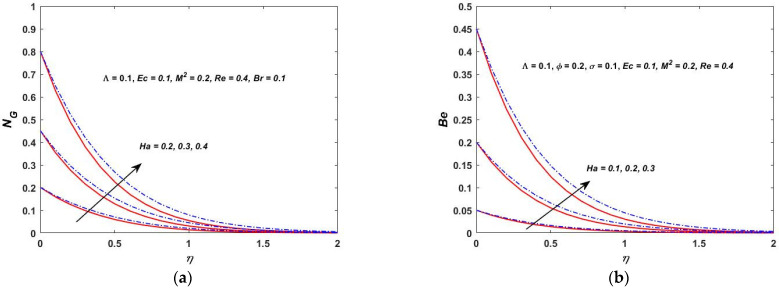
(**a**) Hartmann verses entropy $N_{G}$. (**b**) Hartmann verses Bejan (Be).

As shown in Figure 22a, the temperature difference has a great impact on the entropy generation in the closed and as well in the open system. When the temperature difference parameter is increased, the entropy production is reduced. This indicates that lowering the operating temperature can reduce the entropy production. As can be observed in Figure 22b, the Bejan number decreases as the value of Λ is increased. The heat transfer effects are also observed to be completely dominant at the surface of the rotating stretching disk. As Λ increases, the irreversibility of the fluid friction is increased. This is based on the fact that the viscous dissipation parameter enhances with the lowering operating temperature. Figure 23a,b depict the behavior of $N_{G}$ and the Bejan number when the Br is varied. According to Figure 23a,b, the rapid expansion of the Brinkman number significantly increases the irreversibility of the thermal energy in relation to the entropy generation and the Bejan number. The effect of the Biot number on $N_{G}$ and the Bejan number is depicted in Figure 24a,b. On the rotating stretching disk surface, it was observed that higher values of σ resulted in higher thermal energy irreversibility. The enhanced $N_{G}$ and Bejan number cause an increase in the dominating effects of the fluid friction and heat transfer. In Figure 25a,b, the influence of the rotation parameter S can be seen. The rapid expansion of the Hartmann number, as shown in Figure 26a,b, significantly increases the irreversibility of the thermal energy in relation to the entropy generation and the Bejan number. It was revealed that the increase in S enhances both $N_{G}$ and Bejan number. As a result, all of these characteristics give a good understanding of how to calculate the thermal energy irreversibility in the boundary layer thickness. 

## 6. Conclusions

In this study, two numerical approaches, Keller box and shooting, are used to simulate the three-dimensional carbon nanotube flow on a rotating stretching disk through a convective boundary condition. The following are the key findings:

- With an increase in the values of the regulating flow variables, such as the magnetic parameter, the velocities $f^{\prime }\left(\eta \right)$ and g(η) for both the SWCNTs and the MWCNTs decrease.
- The increase in the rotation parameter causes an increase in velocity $f^{\prime }\left(\eta \right)$ and velocity g(η) in both types of carbon nanotubes.
- As the values of the nanoparticle volume fraction, magnetic number, Eckert number, and Biot number increase, the temperature (θ) of the fluid increases for both the SWCNTs and the MWCNTs, although inverse behavior is observed against the Prandtl number.
- The skin friction coefficient is improved for both forms of CNTs by the increasing values of the parameters S and $M^{2}$.
- As the Prandtl number and the rotation are increased, the heat transfer rate of the fluid increases for the SWCNTs and the MWCNTs, whereas the contrary trend is shown for the magnetic parameter and the Eckert number.
- With increasing magnitudes of rotation S, the Brinkman number Br, the Biot number, the Hartmann number, the entropy, and Bejan number increase significantly in the immediate vicinity of the rotating and stretching disk.
- When the temperature difference is increased, the entropy generation is decreased.
- The current work can be extended for entropy analysis over a magnetized axially rotating stretching disk to save the surface from excessive heating.

## Figures and Tables

**Table 1:** Characteristics (thermophysical) of water and nanofluid.

Properties	Water (Base)	SWCNT	MWCNT
$\rho \left(\mathrm{kg}/m^{3}\right)$	997.1	2600	1600
$c_{p}\left(J/\mathrm{kgK}\right)$	4179	425	796
k(W/mK)	0.613	6600	3000

**Table 2:** Comparison of radial and azimuthal velocities and heat transfer rate at Ec = ϕ = 0 and Pr = 1.

		$f^{\prime }\left(0\right)$		$-g^{\prime }\left(0\right)$		$-\theta^{\prime }\left(0\right)$	
M	S	Mustafa [36]	Present	Mustafa [36]	Present	Mustafa [36]	Present
0	0	−1.1737207	−1.1737207	0.0000000	0.00000000	0.8519914	0.8519914
	1	−0.9483137	−0.94831384	1.4869526	1.4869526	0.8756621	0.8756619
	2	−0.3262439	−0.3262440	3.1278281	3.1278281	0.9304111	0.9304107
	5	3.1937329	3.1937329	9.2535411	9.2535411	1.1291404	1.1291401
	10	12.7208997	12.7208997	22.9134072	22.9134071	1.4259266	1.4259262
	20	40.9056723	40.9056723	60.0129305	60.0129300	1.8944305	1.8944300
2	0	−1.8304896	−1.8304896	0.00000000	0.00000000	0.7260865	0.7260865
	1	−1.6634525	−1.6634527	2.0239449	2.0239445	0.7422122	0.7422118
	2	−1.1753470	−1.1753473	4.1134938	4.1134935	0.7853654	0.7853649
	5	1.89294547	1.89294550	11.1405994	11.1405991	0.9802851	0.9802846
	10	10.8333844	10.8333846	25.7225551	25.7225549	1.2992208	1.2992203
	20	38.1879775	38.1879779	64.0604500	64.0604446	1.7973154	1.7973148

## Data Availability

Data are available upon request.

## Nomenclature

a	Strain rate	*S*	Rotation parameter
$k_{1}$	Porosity	*Pr*	Prandtl number
(u, v, w)	Velocity componants in radial, azimuthal, and axial directions	(r, φ, z)	Cylindrical coordinates in radial, azimuthal, and axial directions
$k_{nf}$	Nanofluid thermal conductivity	Q	Heat flux
Ω	Angular velocity	$c_{p}$	Specific heat
*Re*	Reynold number	$h_{f}$	Convective heat transfer coefficient
$C_{f}$	Skin friction coefficient	$T_{f}$	Heated fluid temperature
$T_{\infty }$	Ambient fluid temperature	$k_{f}$	Fluid thermal conductivity
Greek variables			
$\nu_{nf}$	Nanofluid kinematic viscosity	$\nu_{f}$	Fluid viscosity
$\mu_{nf}$	Nanofluid dynamic viscosity	$\mu_{f}$	Fluid dynamic viscosity
$\beta^{*}$	Non-dimensional thermal relaxation time	$\lambda^{*}$	Dimensional thermal relaxation time
${\left(\rho c_{p}\right)}_{nf}$	Heat capacity of nanofluid	$(\rho c_{p)}{}_{f}$	Heat capacity of fluid
${\left(\rho c_{p}\right)}_{CNT}$	Heat capacity of carbon nanotubes	σ	Biot number
$\alpha_{nf}$	Thermal diffusion of nanofluid	$\rho_{CNT}$	Density of carbon nanotubes
